# Physicochemical and Sensory Properties of *Arabica* Coffee Beans of Arara cv. Dried Using Different Methods

**DOI:** 10.3390/foods13050642

**Published:** 2024-02-21

**Authors:** Emerson G. Coelho, Pedro L. L. Bertarini, Matheus S. Gomes, Laurence R. Amaral, Marta F. Zotarelli, Líbia D. Santos, Ricardo C. Santana

**Affiliations:** 1 Faculty of Chemical Engineering, Federal University of Uberlândia, Patos de Minas 38700-002, Minas Gerais, Brazil; emersongcoelho@hotmail.com (E.G.C.); martazotarelli@ufu.br (M.F.Z.); libia@ufu.br (L.D.S.); 2 Faculty of Electrical Engineering, Federal University of Uberlândia, Patos de Minas 38700-002, Minas Gerais, Brazil; bertarini@ufu.br; 3 Institute of Biotechnology, Federal University of Uberlândia, Patos de Minas 38700-002, Minas Gerais, Brazil; matheusgomes@ufu.br; 4 Faculty of Computation, Federal University of Uberlândia, Patos de Minas 38700-002, Minas Gerais, Brazil; laurence@ufu.br

**Keywords:** coffee drying methods, Arara cultivar, characterization of dried coffee, coffee quality, specialty coffee

## Abstract

The coffee fruit is preferably harvested at the cherry stage, with high moisture and metabolic activity, and must then undergo a drying process for better preservation of the bean and its sensory attributes. In this context, this study aimed to characterize the final quality of the Arara cultivar *Arabica* coffee processed using the wet method and subjected to six drying methods: three conducted at the agro-industrial establishment (fixed-bed dryer, rotary drum dryer, and combined drying) and three laboratory-scale methods (convective oven, cast-tape drying, and suspended terrace). Drying was carried out to reduce the coffee’s moisture content from an initial value of 46.2% on a wet basis (w.b.) to a final average value of 11.35% (w.b.). The fruits of in natura demucilaged coffee and the processed dry coffee beans were characterized for moisture, ash content, nitrogen compounds, lipids, total titratable acidity, organic acids, sugars, and the instrumental color of the beans. The sensory profile of the *Arabica* coffee was evaluated by five coffee specialists using the methodology proposed by the Specialty Coffee Association (SCA), and all the coffees were classified as a specialty.

## 1. Introduction

Coffee is the second-most traded commodity in the world (only after oil), being cultivated in more than 52 countries. It is the world’s most popular beverage and has a strong economic, social, and cultural impact. In 2020, about 169 million 60 kg bags of coffee were produced worldwide, corresponding to more than 3 billion cups [1]. Brazil is the world’s largest producer and exporter of fresh coffee, and coffee is one of its vital productive social and economic activities due to the number of jobs created and the significant contribution to its foreign exchange earnings. The second survey of the 2023 Brazilian coffee crop, a cycle of negative bienniality, estimates a total production of 54.743 million 60 kg bags, which is expected to be 7.5% higher than the 2022 harvest [2]. The Brazilian coffee crop in 2022 amounted to 50.92 million 60 kg bags with a production of 27.7 bags per hectare (bags/ha), and of this total, 80.52% were exported [2].

Despite various coffee species of the genus Coffea (*Rubiaceae* family), only two are widely traded for consumption: *Coffea canephora* and *Coffea arabica* L., commonly known as Robusta coffee and *Arabica* coffee, respectively. The beans of Robusta coffee have a more bitter and stronger flavor than those of *Arabica* coffee, and they are a species of lower commercial value. *Arabica* coffee, in addition to having a higher commercial value, produces a higher-quality beverage with an intense aroma and is most desired by consumers [3]. The Arara cultivar features yellow fruits and exhibits high resistance to rust. It yields high productivity with uniformly sized plants and large fruits. These fruits mature late and produce a good-quality beverage. The cultivar is quite tolerant to drought, making it suitable for denser plantings and warmer locations [4,5].

The excellence of Brazilian coffee, coupled with the demands of international markets, has spurred increased production of specialty coffees. This term refers to coffees with the best flavors produced in unique microclimates under standards and quantifications that allow comparisons between different high-quality coffees. For this purpose, the Specialty Coffee Association (SCA) protocol, which is globally accepted and the most well-known for classifying quality coffees [6], is employed.

At the time of harvest, coffee, depending on its ripeness, can have a moisture content ranging from 30 to 65% on a wet basis (w.b.). This moisture level leads to rapid deterioration due to fermentation, respiration, oxidation, and contamination via microorganisms that thrive in the presence of water, thus compromising the quality of the coffee. Therefore, drying processes are essential to reduce this percentage before storage. After drying, the moisture content of the coffee is reduced to an adequate range of 11 to 12% (w.b.) [7,8].

Coffee is generally dried using natural methods, such as solar energy on drying patios [9] or mechanical dryers with hot air, like fixed beds and rotary dryers. Combinations of these methods can also be used, where the coffee is initially exposed to the sun and then subjected to mechanical drying, typically at temperatures below 40 °C. This is because various studies have shown that higher temperatures can damage the quality of the coffee [10,11]. Drying at temperatures above 40 °C can lead to physical and chemical damage to the coffee beans, resulting in loss of aroma and flavor, weight loss, and deterioration of the quality of the final product [12].

The drying process aims to preserve coffee during storage and should be conducted under suitable operational conditions [13]. This ensures that the product is microbiologically safe and that the dried coffee has the desired uniform physicochemical and sensory characteristics. It is considered a critical operation in the sequence of post-harvest activities and can lead to deterioration of quality and loss of product value if poorly conducted [14]. Therefore, the continuous study of different coffee drying methods is pertinent. This involves evaluating equipment and drying conditions that facilitate moisture reduction with minimal undesirable changes in the properties of the beans. In other words, it is highly relevant to investigate equipment and methods that can improve the coffee drying process. This includes minimizing thermal damage to the beans’ chemical, physical, and sensory characteristics while integrating technology to enhance quality.

Among the conventional methods of drying coffee, natural drying on patios involves spreading the coffee over a surface (which can be made of different materials) to expose the product directly to solar radiation and ambient air convection. Here, the beans are periodically stirred to maintain uniform drying. Modifications to this method aim for improved results, and some configurations include a forced ventilation system with heated air and a suspended patio setup [8]. In most fixed-bed dryers, air flows from the lower layer to the surface of the grain mass. The air/grain moisture exchange occurs in the drying zone, developing two gradients: one of moisture and another of temperature, established between the two layers [15]. The rotary type of dryer consists of a cylinder rotating around its longitudinal axis at a certain angular velocity, which facilitates the movement of the grains during the process. The drying airflow is injected into the chamber located at the center of the cylinder and passes radially through the grain mass [16,17].

Drying in a ‘cast-tape dryer’ (also known as a refractance window dryer) is considered an innovative or non-conventional method and has proven to be very promising when applied to various foods, especially in paste-like form. It involves using heated water (or steam) in contact with the lower face of a flexible support, typically a thin polymeric film (usually polyester), to transfer heat and dry the food distributed on the film’s upper face. This method is predominantly conductive [18]. It has been applied to the drying of various products, but in the case of coffee, its use has been reported in the literature only to produce instant coffee [19].

To date, few articles have been published on the physicochemical characterization and sensory analysis of coffee subjected to different drying methods, particularly non-traditional methods such as cast-tape drying. In this context, this study aimed to investigate the effects of drying *Arabica* coffee beans (*Coffea arabica* L., Arara cultivar from the Cerrado Mineiro region of Brazil) by analyzing their physicochemical and sensory characteristics. This was conducted for beans both in their natural state and when subjected to different methods and equipment on an industrial scale (fixed-bed dryer, rotary drum dryer, and combined drying (fixed bed followed by rotary drum)) and on a laboratory scale (convective oven, cast-tape drying, and suspended terrace). Besides identifying the best operational conditions for traditional methods, such as natural drying, convective drying with forced hot air ventilation, and mixed methods, there is significant interest in researching unconventional methods for coffee, like cast-tape drying, to enhance the quality of the beans and add economic value to the product.

## 2. Materials and Methods

### 2.1. Coffee

The coffee samples (*C. Arabica* L., Arara cultivar) were collected from the same field, AR171, planted in 2017 at Fazenda Chuá. This farm is located in Patos de Minas, Minas Gerais, Brazil, at latitude 18°35′15″ S, longitude 46°25′35″ W, and an altitude of 1050 m. The fruits were mechanically harvested at the cherry stage in July 2021 and processed on the farm, where they were washed and pulped.

The cherry coffee was washed to remove dirt and impurities, then separated by density difference (to remove the overripe fruits) and processed using the wet method. The exocarp and mucilage were mechanically removed in this process, resulting in demucilaged cherry coffee (CD). Before these beans underwent the drying operation, they were in their natural, demucilaged state when a 100 g sample of the coffee in its natural state was preserved in an ultrafreezer (Indrel, model IULT335D, São Paulo, Brazil) at −60 °C for subsequent analysis to characterize the fresh coffee fruit. This process is more complex and tends to yield coffee with a smoother and more complex flavor. After drying and further processing, the processed dry coffee beans were obtained.

### 2.2. Coffee Drying

**Figure 1:**
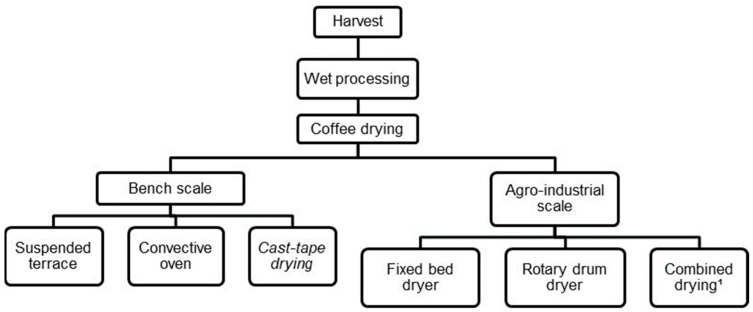
Flowchart of coffee processing and drying methods. ^1^ Pre-drying in a fixed-bed dryer followed by drying in a rotary drum dryer.

The drying methods studied are depicted in Figure 1. The drying operations, both at the bench scale (laboratory) and agro-industrial scale, were carried out intermittently, with the coffee being dried during the day and left to cool/rest overnight.

In the bench-scale experiments, suspended terrace drying, convective oven drying, and cast-tape drying were employed. For agro-industrial drying, the processed coffee underwent drying using forced hot air in a fixed-bed dryer (Penágos, Ecodrying SED 15, Três Pontas, Brazil), a rotary drum dryer (PaliniAlves, PA-SR/15, Espírito Santo do Pinhal, Brazil), or a combination of these two dryers, starting with the fixed-bed dryer for pre-drying, followed by the rotary drum dryer.

The suspended terrace was constructed using hexagonal metallic wire mesh of 18 BWG with a 1.24 mm diameter and 15 mm mesh, complemented by a 50% Sombrite^®^ shade cloth. It was placed in a ventilated and sunny location. For the drying process, 8 L of demucilaged coffee was spread on the surface of the suspended terrace, forming a layer approximately 3 cm thick.

Another 8 L sample of demucilaged coffee was used for drying in a convective oven (Ethik Technology, Model 400-8D, 2012, Vargem Grande Paulista, Brazil). To carry out the drying using this method, the coffee was spread in a 3 cm thick layer on the surface of an expanded sheet tray. This tray featured holes across its extent, allowing the passage of the heated airflow.

**Figure 2:**
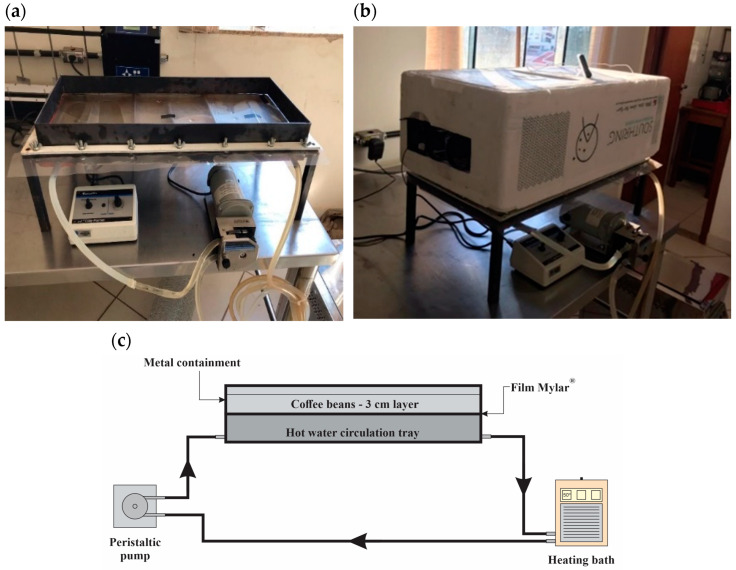
Cast-tape drying: (**a**) experimental cast-tape dryer; (**b**) cast-tape dryer coupled to the exhaust system; and (**c**) operational scheme of the cast-tape dryer.

In the laboratory, an 8 L sample of demucilaged coffee was designated for drying in a cast-tape dryer (shown in Figure 2). The custom-built dryer consisted of a stainless-steel tray measuring 70 cm × 30 cm × 5 cm. Hot water, supplied from a thermostatic bath (SOLAB, model SL 152/18, Piracicaba, Brazil) and circulated using a peristaltic pump (Cole-Parmer, model Masterflex L/S 77250-62, Vernon Hills, IL, USA), flowed through this tray. A polymeric film (Mylar^®^ type ‘D’ (Dupont, Chester, VA, USA), 0.25 mm thick) was affixed on top of the tray, with its lower face in contact with the circulating hot water and the upper face supporting the coffee beans. An exhaust system was integrated into the equipment, constructed using a box of expanded polystyrene with six fans installed: three to move external air into the equipment and three to expel internal air into the external environment. This system provided airflow over the coffee layer to remove the stagnant air layer during drying.

The used fixed-bed dryer was an Ecodrying SED 15, with a capacity of 15,000 L distributed over three floors; it features automated and programmable turnings and is powered using a 24 hp electric motor. The dryer consists of a heat exchanger set, which receives water heated using a wood-fired furnace. The heated water circulates through the radiator, and a fan blows air that is heated via convection. The coffee beans are placed on the three floors, where the cross-flow of heated air passes through the coffee mass, combined with periodic turnings, which facilitates relatively quick and uniform convective drying.

The rotary drum dryer (PA-SR/15) used has a capacity of 15,000 L, with drum dimensions of 5.67 m in length and 2.21 m in diameter, and is equipped with a 5 hp electric motor. The dryer consists of a set formed by the drying cylinder (drum), fan, and radiator, which receives hot water from a wood-fired hot water boiler. The coffee introduced into the dryer is subjected to convective drying through contact with the hot air stream.

In the combined drying method, the demucilaged coffee was first placed in the feed hopper of the Ecodrying SED 15 fixed-bed dryer. Here, pre-drying was conducted until the moisture content reached 25% (w.b.), a value experimentally determined to equalize the drying times and thus utilize both pieces of equipment effectively. After the pre-drying, the coffee was transferred to the PA-SR/15 rotary drum dryer, where the drying process was completed.

After the agro-industrial-scale drying processes, the beans were transferred to a storage bin, where they remained for one day to homogenize the moisture content. They were then processed and stored in raffia bags in a closed area at room temperature. Following a 30-day resting period, the beans underwent secondary processing to remove the endosperm (parchment). This hulling was performed in two passes through a coffee sample huller. Afterwards, the material was sifted to remove the chaff. The cleaned beans were subsequently used for analyses and characterizations.

In order to study the drying kinetics of the methods used at the laboratory scale (suspended terrace drying, convective oven drying, and cast-tape dryer drying), random samples of 3 to 5 g of beans were collected in triplicate from each of the three methods. These samples were taken every two hours on the first day and then every five hours on subsequent days. Moisture was determined using the gravimetric method according to the methodology proposed by the AOAC [20]. This involved drying the grains in an oven at 105 °C for 24 h. After removal from the oven, the crucibles were cooled to room temperature in a desiccator and then weighed on an analytical balance. The analyses were performed in triplicate. The results were expressed in % (w.b.) (grams of water per 100 g of sample). Due to difficulties and sampling restrictions in the agro-industry, it was not possible to perform the drying kinetics study of coffee using the agro-industrial methods.

### 2.3. Coffee Physical–Chemical Characterization

For the characterization of the in natura coffee fruits and coffee beans subjected to the 6 drying methods, the samples were prepared with 300 g of in natura demucilaged coffee fruits and 300 g of processed dry coffee beans for each drying method. These samples were freeze-dried for 48 h at 214 mmHg and a temperature of −60 °C. After freeze-drying, the beans were ground in a knife mill to a particle size of −20 mesh [21].

The determination of the moisture content of the coffee beans was carried out according to the methodology proposed by the AOAC [20], which involves weighing 3 to 5 g of coffee beans in previously dried and tared crucibles and subjecting these beans to drying in an oven at 105 °C for 24 h. After removal from the oven, the crucibles were cooled to room temperature in a desiccator and weighed on an analytical balance.

The method used to analyze ash content was incineration in a muffle furnace, in which all organic matter is burned. For this, 3 to 5 g of coffee beans were weighed, which were placed in a porcelain crucible with a previously established mass and kept in the muffle furnace at 550 °C for 6 h or until only ash remained in the crucible according to the method described by the AOAC [20].

The content of nitrogen compounds and the total protein content were determined using the adapted Macro-Kjeldahl method, as described in the AOAC methodology [20].

The ether extract was determined via extraction with petroleum ether in a Soxhlet apparatus, as described in the AOAC methodology [20]. Approximately 2 g of coffee samples were wrapped in filter papers to form a closed cartridge. The cartridges were placed in tubes, which were then fitted into the Soxhlet apparatus and subjected to 6 h of heating at a temperature of 60 °C for lipid extraction.

The total titratable acidity was determined after rehydration of the freeze-dried and ground samples (2 g of sample in 50 mL of distilled water). The suspensions were titrated with a 0.1 N NaOH solution until reaching a pH of 8.3 according to the method described by the AOAC [20].

The instrumental color of the processed dry coffee beans was determined using a colorimeter (Minolta model DP-400, Ramsey, NJ, USA) by direct reading of the L*, a*, and b* coordinates, as described by De Almeida Couto [22]. Before each measurement, the instrument was calibrated on a white tile (L* = 98.82; a* = −0.18; and b* = −0.31).

### 2.4. Identification of Organic Acids, Sugars, and Alcohols

Organic acids, sugars, ethanol, and glycerol were analyzed using a high-performance liquid chromatography (HPLC) system (Shimadzu, model LC-20A Prominence, Tokyo, Japan, using a SUPELCOGEL C610H column) according to methods described by Ali [23]. The sample was diluted, filtered, and injected into the chromatographic system, where the components were separated and detected via light refraction. The mobile phase used was deionized water, the pump flow rate was 0.5 mL/min, the oven temperature was 32 °C, and the injection volume was 20 µL.

### 2.5. Sensory Analysis

The samples were prepared according to the methodology of the Specialty Coffee Association [24]. A total of 150 g of *Arabica* coffee from each drying method was roasted in a sample roaster to achieve a medium roast. The beans were ground in a coffee grinder, obtaining 70 to 75% of the particles at a 20-mesh size.

A panel of five trained coffee specialists with Q-Grader Coffee Certificates evaluated the samples. The methodology to assess the coffees was conducted according to SCA standards, which evaluate ten sensory attributes: fragrance, flavor, aftertaste, acidity, body, uniformity, balance, sweetness, cleanliness, and overall.

All determinations were made in triplicate, except for the sensory analysis (quintuplicates) and the instrumental color analysis of the beans (10 replicates). The averages of the physicochemical characterization analysis of coffee and the sensory analysis data were subjected to ANOVA, followed by a post hoc comparison of the means using the Tukey test with a 5% significance level in the hypothesis testing.

## 3. Results and Discussion

### 3.1. Drying Kinetics

According to the literature [25,26], it is well established that to achieve a beverage with quality physicochemical and sensory attributes classified as special, coffee drying should be conducted in a manner that maintains the grain temperature below 40 °C and yields a dry product with a moisture content of around 12%. The drying operations were carried out to ensure the coffee beans reached this ideal moisture level for safe storage without compromising the quality of the coffee.

In this context, drying on the suspended terrace took 5.5 days to reach a moisture content of 11.1% (w.b.), totaling 125 h. This included 55 h of solar exposure and 70 h of coffee covered with a tarp at night. However, as shown in Figure 2, it is important to note that between 105 and 120 h of operation on the suspended terrace, the grains had already achieved a moisture level of around 12%. In the convective oven, the coffee reached 11.3% (w.b.) moisture by the 5th day of drying, amounting to 96 h of experiments. This included 40 h with the oven heated to 40 °C and 56 h with the oven turned off and closed. In the cast-tape dryer, the coffee reached a moisture content of 11.2% (w.b.) on the 5th day as well, after 101 h, with 55 h of the system operating and 46 h of the coffee cooling and resting with the system turned off.

**Figure 3:**
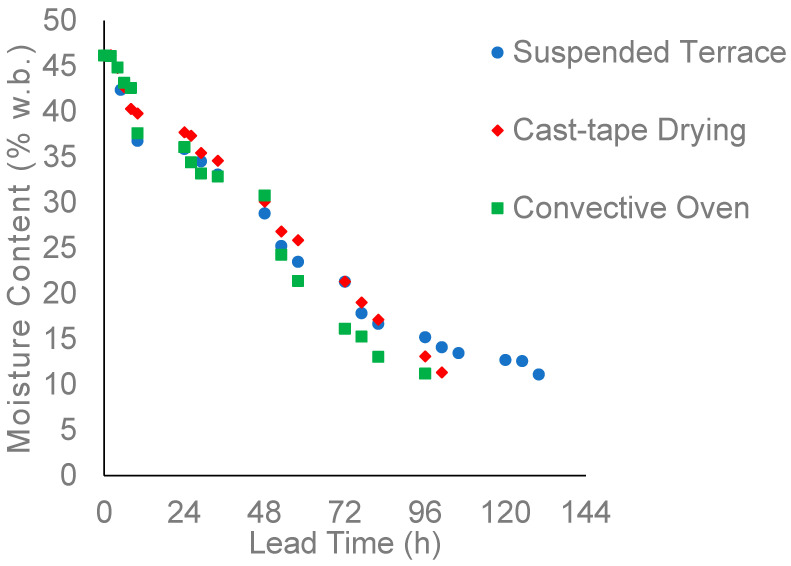
Drying kinetics of *Arabica* coffee dried in the suspended terrace, convective oven, and cast-tape dryer.

The in natura demucilaged coffee fruits, that is, prior to the drying operations, had a moisture value of 46.2 ± 0.49% (w.b.). Figure 3 presents the results of the drying kinetics of *Arabica* coffee dried in the suspended terrace, convective oven, and cast-tape dryer.

It was noted that to achieve coffee with approximately 12% moisture content, drying on the suspended terrace required a longer operating time to reach this desired value, possibly due to temperature variations throughout the day. In contrast, in the convective oven, the shortest time was observed among the methods implemented on a laboratory scale. This is likely attributable to better control over operating conditions, such as temperature and air circulation.

In the study by Coradi and Borém [27], the drying of demucilaged coffee on suspended terraces took place over 8 days, with two turnings per hour during the day (8 h/day). On the other hand, Ribeiro et al. [28] found that drying pulped coffee using suspended terraces reached 11 to 12% moisture content in 284 h (10 days). Kitzberger et al. [29] determined that cherries with similar ripeness (70–90% ripe fruits) and selected only ripe fruits were dried naturally on sun terraces, reaching a moisture content of approximately 12% between 15 and 21 days.

As mentioned previously, it was not possible to study the drying kinetics using industrial methods. However, it took 6 days (144 h), with approximately 58 h, with the fixed-bed dryer running to reach a moisture content of 11.2% (w.b.). De Oliveira et al. [9] reported in their study a fixed-bed dryer adapted for drying a volume of 15,000 L of pulped *Arabica* coffee in 132 h, corroborating the results of the present study. In the rotary drum dryer, the coffee was dried to a moisture content of 11.5% (w.b.) over 5 days (120 h), with 52 h of the dryer in operation. Combined drying occurred over 6 days (144 h), following a pre-established procedure already defined as the industry standard for the investigation of drying, with 2.5 h in the fixed-bed dryer and 47 h in the rotary dryer, reaching 11.4% (w.b.). However, a proper comparison of drying times between industrial and laboratory methods is not possible due to the difference in scale.

### 3.2. Physical and Chemical Analysis of Coffee

#### 3.2.1. Moisture, Ash, Nitrogen Compounds, and Total Titratable Acidity

The average values of moisture (w.b.), ash content, nitrogen compounds, lipids, and total titratable acidity of the fresh demucilaged coffee fruits and the processed coffee beans subjected to different drying methods are summarized in Table 1.

The initial moisture content of the fresh mucilage-free coffee fruits was around 46% (w.b.) and was reduced through drying methods to between 11.1 and 11.5% (w.b.). It is worth noting that the fact that the final moisture content in all methods is the same does not necessarily mean that the equilibrium moisture content was reached under the investigated conditions. In this way, it was expected that the final moisture values found would be significantly similar, as verified.

No statistically significant differences were observed in the ash content among the different drying processes, neither between the demucilaged coffee nor the dried coffees. This indicates that heat exposure did not affect this property. Notably, the results are below the maximum limit established by the National Health Surveillance Agency [30], recommending values below 5% for ground and packaged coffees. In this study, the ash contents (refer to Table 1) were higher than the average value of 3.44% found by Fernandes et al. [31] in a study comparing *Arabica* and Robusta coffees from different harvests and a blend containing 70% *Arabica* and 30% Brazilian Conilon coffee. The results obtained are also close to those mentioned by Clarke [32], who reported values between 3.8% (*Arabica* coffee) and 4.14% (Robusta coffee) on average for 42 samples. These values also align with findings reported by Pittia et al. [33] and Jokanović et al. [34], which found values between 3.55 and 4.06% for roasted *Arabica* coffees.

Significant differences were found among some drying processes in the determination of nitrogen compounds. The highest concentrations were obtained in laboratory processes (cast-tape dryer and convective oven) and a fixed-bed dryer—with statistically equal values among themselves and concerning the concentration of naturally demucilaged coffee. The lowest concentrations were found in natural drying on suspended patios on a laboratory scale and in agro-industrial-scale drying methods involving rotary dryers, with no significant differences in values. The observed reduction in nitrogen compounds across various drying methods can be attributed to the interplay of time and temperature. Elevated temperatures coupled with extended drying durations can precipitate phenomena such as protein denaturation, thermal decomposition of nitrogenous compounds, and oxidative reactions. The higher the protein levels in the beans, the greater the possibility of aromatic compound formation during roasting [35]. Additionally, they are also responsible for the color formation of the beverage [36,37,38]. The percentages in the cited literature are higher than those in the present study. Geromel et al. [39] and Scholz et al. [38] observed nitrogen compound values ranging from 14.5% to 17.0%, respectively, for naturally sun-dried modern and traditional Brazilian *Arabica* coffee cultivars on patios.

The values obtained for lipid composition were statistically equal among the drying methods (*p*-value > 0.05) and higher compared to naturally demucilaged coffee fruits. This fact indicates that the individual analysis of this characteristic is not a variable that will interfere with the choice of the drying method. The lipid values found were within the limit for dried *Arabica* coffees, as presented by Pereira et al. [40]. In the study by Kitzberger et al. [29], lipid values between 12.0 and 14.4% were found for *Arabica* coffees of different cultivars and geographical origins in Brazil, while in the work of Oliveira et al. [41], values around 16% were found for *Arabica* coffees from the Cerrado Mineiro and South of Minas Gerais (Brazil) after drying on asphalt patios. Both presented lipid values are close to those found in the present study. According to Dias et al. [4], lipids represent between 12 and 18% of the bean’s dry weight, confirming this study’s results as well.

The quantified values for total titratable acidity were statistically equal when comparing all drying methods. The results obtained fall within the range (though close to the upper limit) of the results presented by Malta, Chagas, and Oliveira [42] for *Arabica* coffees (average values of different cultivars), which ranged from 233.33 to 300 mL of 0.1N NaOH per 100 g. On the other hand, they are higher than those found for *Arabica* coffees by Lopes, Pereira, Mendes [43] (average values of different cultivars), and Reinato et al. [44], ranging from 220.00 to 225.00 mL of 0.1N NaOH per 100 g. When compared to the in natura sample, the coffees dried using the various investigated methods showed statistically lower values at a significance level of 5%. Therefore, it can be said that all methods tend to reduce the total titratable acidity value, which could result in desirable acidic nuances in the beverage’s taste.

#### 3.2.2. Organic Acids

Table 2 presents the values of organic acid content (citric, malic, and succinic acids) in green beans, both in their natural state before and after being subjected to drying. The concentrations are expressed in milligrams of organic acid per gram of coffee on a dry basis.

In terms of sensory quality, the acidity of coffee is an essential attribute. Acidity resulting from citric, malic, and succinic acid levels contributes to desirable acidity for coffee quality. In contrast, acidity from acetic, butyric, and propionic acids is undesirable for coffee quality [45]. A well-balanced acidity, combined with aromatic, fruity notes and a subtle aroma of citrus and almond, is often appreciated as a significant characteristic of high-quality coffee [46]. This associates the acidity of organic acids with superior coffee quality [47].

The citric acid content in the sample of naturally demucilaged coffee was statistically equivalent to the values quantified using the different drying methods. It was also observed that the value for citric acid content in coffee dried using industrial combined dryers (10.40 mg·g^−1^ of dry coffee mass) was lower than those identified in coffee dried on suspended patios, fixed-bed dryers, and rotary drum dryers. However, it was statistically similar to the values found in other drying methods (convective oven and cast-tape dryer). In the study by Ribeiro et al. [28], citric acid values for wet-processed *Arabica* coffee, without fermentation, using only ripe cherries and naturally demucilaged, were 5.4 mg·g^−1^ of dry coffee mass (Cultivar Ouro Amarelo) and 7.5 mg·g^−1^ of dry coffee mass (Cultivar Mundo Novo). These values were lower than the result found in the Arara coffee in this study, which may be attributed to the exclusive use of ripe cherries.

Similarly, it was observed that there was no statistically significant difference between the malic acid content found in the sample of naturally demucilaged coffee and the values detected in the different drying methods. Regarding comparisons among the various drying methods, it is noted that the value in combined drying, i.e., fixed-bed drying followed by rotary drum drying (3.68 mg·g^−1^ of dry coffee mass), was statistically lower than the result quantified in coffee dried on suspended patios and equivalent to the values obtained in the other dryers. These results are aligned with those found by Kitzberger et al. [29], which ranged from 3.0 to 5.4 mg·g^−1^ for dried demucilaged *Arabica* cherry coffee on suspended patios. In the study by Ribeiro et al. [28], malic acid values were found to be 1.3 mg·g^−1^ of dry *Arabica* coffee mass (Cultivar Ouro Amarelo) and 1.5 mg·g^−1^ of dry *Arabica* coffee mass (Cultivar Mundo Novo) for wet-processed and fermentation-free coffees dried on suspended patios. Pereira [21] reported not finding malic acid concentrations in the *Arabica* Catuaí cultivar of pulped coffee and sun-dried coffee without fermentation.

The succinic acid levels found in all drying methods were statistically similar. However, they differed when compared to the in natura demucilaged coffee sample, in which the content of this acid was higher (2.37 mg·g^−1^ of the coffee sample) before undergoing the drying operation. In the study by Bressani et al. [48], concentrations of succinic acid of 0.40 mg·g^−1^ were found in cherry *Arabica* coffee (Catuaí Vermelho) after fermentation-free drying, similar to those found in the present study. In the study by Ribeiro et al. [28], values for control coffees were 2.4 mg·g^−1^ (Cultivar Ouro Amarelo) and 4.9 mg·g^−1^ (Cultivar Mundo Novo) of coffee samples for naturally ripe, depulped, and wet-processed cherry coffee without induced fermentation.

The literature shows that other organic acids, such as acetic, butyric, lactic, and propionic acids, can be part of coffee’s composition [48,49]. However, their identification is often associated with induced coffee fermentation, which, if present in this study, was not intense enough for their occurrence and quantification. This finding is significant because acetic, butyric, and propionic acids can lead to substantial quality drawbacks in beverages [7]. In this study, acetic, butyric, lactic, and propionic acids were not detected, which is consistent with Pereira’s study [21]. Pereira investigated controlled fermentations with freshly harvested cherry coffees (Catuaí cultivar) and did not detect these acids before coffee fermentation but found them afterward. Bressani et al. [48], in their analysis of the chemical composition of sun-dried cherry *Arabica* coffees with yeast inoculation, did not detect lactic and butyric acids at the beginning of fermentation. Ribeiro et al. [28] found acetic acid values of 1.1 mg·g^−1^ of dry coffee mass (Cultivar Ouro Amarelo) in control coffee (without induced fermentation) processed via wet processing and 1.8 mg·g^−1^ of dry coffee mass (Cultivar Mundo Novo) in coffee samples. Pimenta, Costa, and Chagas [37] noted that under anaerobic conditions and/or in the presence of microorganisms, the sugars in the mucilage can ferment, producing alcohol, which then converts into acetic, butyric, and propionic acids, significantly deteriorating beverage quality. However, acetic acid is not harmful if it is present in low quantities.

#### 3.2.3. Sugars and Glycerol

The predominant free sugars in coffee beans are primarily sucrose, fructose, and glucose [50]. Table 3 presents the values of sugar content (fructose, glucose, and sucrose) in green beans, both in their in natura state before and after being subjected to drying, expressed on a dry basis. Contributing to its sweet flavor, which is one of the most desirable attributes of specialty coffees, sugars are closely linked to coffee quality. Higher concentrations of sugars in raw beans can lead to an increase in these components during the roasting process, and there is a crucial relationship between the quantities of non-reducing and reducing sugars and coffee quality. However, there is no consensus in the literature regarding the type and concentration of sugars in beans that influence the quality attributes of the beverage, such as aroma, taste, acidity, bitterness, and color [51].

It was observed that the levels of fructose, glycerol, and glucose significantly decreased after drying, while the sucrose content increased, corroborating the results of Santos et al. [51]. The reduction in reducing sugars, like glucose and fructose, may be attributed to fermentative processes occurring during the drying period [52].

Statistical analysis revealed no significant difference in the fructose content values observed across the different drying methods. In his study with *Arabica* coffee (Catuaí cultivar) dried in a 35 °C air recirculation oven, De Carvalho Neto [53] found a content of 3.5 mg of fructose per gram of dry coffee mass. Similarly, the glycerol content values were statistically consistent.

Regarding the glucose content, no significant difference was observed between samples dried using different processes. However, these values were generally higher than those found by other authors in studies of depulped *Arabica* coffee, which reported average values of 0.22 mg of sugar per gram of dry coffee mass after drying [54]. Glucose, a simple sugar, contributes to coffee’s sweetness. The higher its content, the greater the perceived sweetness of the beverage. This balances the natural acidity of coffee, contributing to a higher score in this attribute.

The sucrose levels found across the different drying methods were statistically equivalent. Knopp, Bytof, and Selmar [55] reported sucrose levels ranging from 50 to 85 mg of sucrose per gram of dry coffee mass for cherry *Arabica* coffee (Acaiá cultivar) dried on patios, which corroborates the findings of this study. According to Borém [15], the sucrose concentration in commercial *Arabica* coffees varies between 50 and 120 mg per gram of dry coffee mass. This range validates the result of this study (68.19 mg of sugar per gram of dry mass).

Murkovic and Derler [54] noted that sugar levels are positively associated with better coffee quality and tend to undergo changes during drying. These compounds play a crucial role in the chemical reactions that occur during coffee roasting, such as the Maillard reaction, which leads to the formation of compounds responsible for the beverage’s characteristic color, aroma, and flavor [8]. The sugar levels found in this investigation were generally higher when compared to other studies, indicating that these sugars contributed to the classification of the coffee as “Special” as determined via a sensory analysis.

#### 3.2.4. Instrumental Color of Coffee Beans

Borém et al. [26] correlated the color of coffee beans with beverage quality, an essential factor in its commercial value. Table 4 presents the instrumental color parameters measured for dried and processed coffee beans subjected to different drying processes.

It was observed that there were no significant differences in any parameter across all methods. Notably, all values on the L* (lightness) parameter scale were around 40. This value ranges from black (0) to white (100) and indicates the lightness or darkness of the coffee beans. A higher L value suggests a lighter color, which might be associated with certain drying methods causing less thermal degradation or browning. C* (Chroma or Saturation) measures the vividness or dullness of a color. In the context of coffee beans, a higher Chroma value might indicate a more intense color. However, this was not observed in this study, according to the results. The Hue angle (h) represents the type of color (such as red, yellow, green, etc.). Variations in the color of green coffee beans can indicate potential natural enzymatic biochemical transformations and oxidative processes that might alter the composition of aroma and flavor precursors in coffee, potentially reducing beverage quality [26]. The h parameter around 88° indicates a tendency towards yellow, but all values show statistically similar results.

Menchú et al. [56] stated that drying in mechanical dryers can alter the color of coffee beans, resulting in unevenly colored masses. Additionally, temperatures above 80 °C can produce grayish beans that undergo irregular bleaching when absorbing water. However, since the coffees in this study were dried at temperatures not exceeding 40 °C, this uneven coloration was not observed, contributing to the production of specialty coffees. Other authors, such as Silva et al. [57] and Corrêa, Afonso Júnior, Pinto [58] also reported that drying alters the color of coffee beans, especially in mechanical dryers. They further asserted that drying on patios, due to lower ambient temperatures, does not significantly affect the color characteristics of beans compared to those dried in mechanical dryers. In the experiments conducted in this study, meticulous temperature control was maintained in both laboratory and agro-industrial drying processes, and as a result, color defects typically generated during drying were not observed.

#### 3.2.5. Sensory Analysis

**Figure 4:**
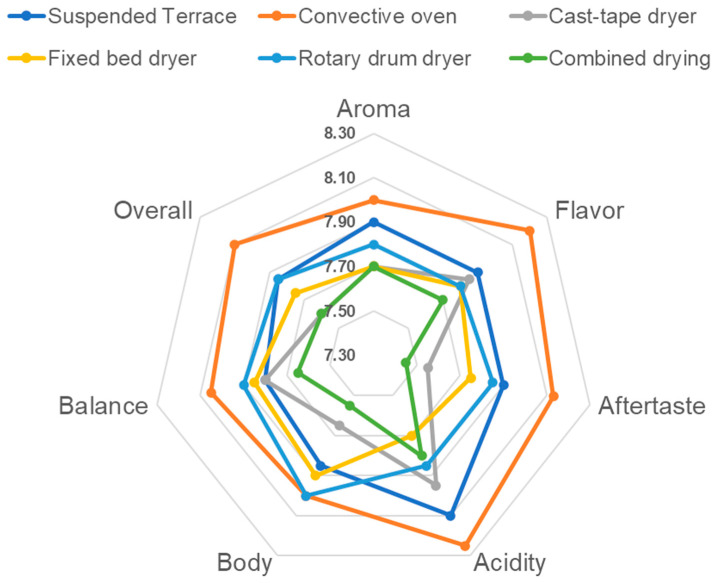
Sensory profile of coffee dried via different drying processes.

The results of the scores assigned to coffees subjected to the different drying methods studied, regarding the main attributes and total score of sensory analysis, are shown in Table 5 and Figure 4. The scores for cup cleanliness, uniformity, and sweetness were all 10 in all conditions and, therefore, are not presented in Table 5.

Through sensory analysis, the coffee dried via different drying processes obtained through the six different methods was classified as ‘Special’, with scores ranging from 83.45 to 86.75 on the SCA scale. The drying methods on suspended terraces, convective dryers, and rotary mechanical dryers resulted in coffees classified as ‘Excellent’. According to Cesar [6], these coffees achieve a total score between 85.00 and 86.75. Considering the standard deviation, even the coffees produced using other methods may have received scores very close to or higher than 85.00. The flavor and aroma attributes of coffee dried in the convective dryer tended to be superior to the others, achieving a final score of 86.75 points, thus defining this coffee as ‘Excellent’ with a high market value. However, the results did not differ statistically and could have been equal to or greater than a score of 85.00, demonstrating the quality of the products obtained and suggesting that the best drying method/equipment could be selected based on a balance between higher quality and shorter drying time.

Although there was no statistically significant difference, generally, the samples evaluated from convective drying tended to have the highest scores in all attributes. Factors contributing to this more positive result include more precise temperature control, continuous airflow, and conditions of this process, such as heat transfer on both sides of the drying product mass. These may have facilitated convective heat transfer in a smaller and better-isolated system than the other methods. This could have resulted in a more homogeneous temperature of the grain mass, aiding in maintaining the quality attributes of the beans. According to Borém et al. [59], careful monitoring of the temperature reached by the beans, minimal movement of the beans, and optimization of the airflow in contact with the coffee layer in convective drying across different equipment are process variables associated with better beverage quality. This may have translated into the observed trend of higher final scores in the convective dryer.

Organic acids, such as citric, malic, and succinic acid, contribute significantly to the sensory experience of coffee, influencing its acidity, aroma, and flavor. The acidity of coffee, a highly valued attribute, is enriched by citric acid, which is associated with citrus and fruity notes that enhance the beverage’s complexity. Malic acid contributes hints of apple or green fruit, while succinic acid adds to the overall complexity of the aroma. In terms of flavor, these acids directly influence the perceived notes, providing a richer and more diverse palate [60].

## 4. Conclusions

Under the operational conditions studied, it can be concluded that, on a laboratory scale, drying on the suspended terrace was the method that required the longest operating time to dry the *Arabica* coffee to reach the required final moisture. However, considering the industrial scale, a longer drying time was observed for fixed-bed dryers and combined dryers. On the other hand, a shorter drying time was obtained for the process in the convective oven dryer, followed by the cast-tape dryer. Regarding organic acid content, citric, malic, and succinic acids were not identified in any dry product sample, indicating no harmful fermentations in the green coffee beans. Citric, malic, and succinic acids were found at levels as observed in the literature. Concerning the color of the dry coffee beans, there was no significant difference when comparing the different drying methods. Also, the results obtained—which encompass characteristics of the coffee from planting through to processing conditions leading to the dry product, among both laboratory-scale drying methods and industrial dryers—demonstrated physicochemical and sensory attributes that resulted in coffee of the same quality classification (specialty). These findings suggest that more comprehensive studies should be conducted, considering factors such as energy consumption and labor costs for each drying method. Consequently, scientific research into coffee drying methods is intensifying to contribute to developing technologies for Brazilian producers that preserve the quality of the product.

## Figures and Tables

**Table 1:** Results of moisture, ash, nitrogen compounds, lipids, and total titratable acidity of demucilated coffee in natura and coffee dried via different drying processes.

	Moisture (% w.b.)	Ashes (%)	Nitrogenous Compounds (%)	Lipids (%)	Total Titratable Acidity (mL NaOH 0.1N∙100 g^−1^)
	Demucilated coffee in natura				
	46.2 ± 0.49 ^a^	3.95 ± 0.04 ^a^	11.3 ± 0.60 ^a^	8.62 ± 0.36 ^b^	303.70 ± 9.90 ^b^
Method	Processed dry coffees				
Suspended terrace	11.1 ± 0.79 ^b^	3.98 ± 0.02 ^a^	7.5 ± 0.60 ^b^	14.00 ± 1.03 ^a^	282.28 ± 2.22 ^a^
Convective oven	11.3 ± 0.87 ^b^	4.01 ± 0.10 ^a^	12.8 ± 1.00 ^a^	14.00 ± 0.29 ^a^	295.07 ± 5.12 ^a^
*Cast-tape dryer*	11.2 ± 0.25 ^b^	3.98 ± 0.07 ^a^	12.0 ± 0.80 ^a^	14.20 ± 0.58 ^a^	280.99 ± 7.57 ^a^
Fixed-bed dryer	11.2 ± 0.79 ^b^	4.03 ± 0.11 ^a^	10.7 ± 1.20 ^a^	14.88 ± 0.42 ^a^	281.00 ± 1.94 ^a^
Rotary drum dryer	11.5 ± 0.87 ^b^	4.00 ± 0.04 ^a^	6.7 ± 1.00 ^b^	14.26 ± 0.25 ^a^	283.96 ± 9.39 ^a^
Combined drying	11.4 ± 0.25 ^b^	4.02 ± 0.03 ^a^	6.7 ± 1.00 ^b^	14.88 ± 0.60 ^a^	279.05 ± 7.79 ^a^

Different letters indicate statistically significant differences between the coffee samples (*p* ≤ 0.05).

**Table 2:** Values of organic acids of demucilated coffee in natura and coffee dried via different drying processes. Data expressed as means ± standard deviation (*n* = 3). Means followed by the same letters in the column do not differ by Tukey’s test at a 5% significance level.

	Citric	Malic	Succinic
	(mg of Acid. g^−1^ of Dry Coffee Mass)		
Demucilated coffee in natura			
	10.86 ± 0.56 ^a,b^	4.04 ± 0.34 ^a,b^	2.37 ± 0.49 ^a^
Method	Processed dry coffees		
Suspended terrace	12.33 ± 0.41 ^a^	4.48 ± 0.13 ^a^	0.59 ± 0.07 ^b^
Convective oven	11.57 ± 0.74 ^a,b^	4.04 ± 0.37 ^a,b^	0.78 ± 0.17 ^b^
Cast-tape dryer	11.87 ± 0.64 ^a,b^	4.04 ± 0.30 ^a,b^	0.68 ± 0.33 ^b^
Fixed-bed dryer	12.36 ± 0.33 ^a^	3.93 ± 0.18 ^a,b^	0.86 ± 0.16 ^b^
Rotary drum dryer	12.19 ± 0.25 ^a^	4.03 ± 0.01 ^a,b^	0.55 ± 0.12 ^b^
Combined drying	10.40 ± 0.72 ^b^	3.68 ± 0.40 ^b^	0.65 ± 0.24 ^b^

Different letters indicate statistically significant differences between the coffee samples (*p* ≤ 0.05)

**Table 3:** Content of fructose, glycerol, glucose, and sucrose of demucilated coffee in natura and coffee dried via different drying processes.

	Fructose	Glycerol	Glucose	Sucrose
	(mg of Sugar g^−1^ of Dry Coffee Mass)			
Demucilated Coffee in natura				
	11.29 ± 0.93 ^a^	1.45 ± 0.35 ^a^	4.27 ± 0.39 ^a^	68.19 ± 3.55 ^a^
Method	Processed dry coffees			
Suspended terrace	5.37 ± 0.37 ^b^	0.22 ± 0.54 ^b^	0.68 ± 0.11 ^b^	84.92 ± 2.03 ^b^
Convective oven	5.04 ± 0.56 ^b^	0.21 ± 0.31 ^b^	0.56 ± 0.01 ^b^	80.87 ± 2.13 ^b^
Cast-tape dryer	4.93 ± 0.68 ^b^	0.19 ± 0.41 ^b^	0.65 ± 0.11 ^b^	79.55 ± 3.20 ^b^
Fixed-bed dryer	4.69 ± 0.37 ^b^	0.26 ± 0.32 ^b^	0.45 ± 0.04 ^b^	78.99 ± 1.02 ^b^
Rotary drum dryer	4.53 ± 0.20 ^b^	0.28 ± 0.66 ^b^	0.41 ± 0.01 ^b^	84.31 ± 1.89 ^b^
Combined drying	4.33 ± 0.49 ^b^	0.25 ± 0.48 ^b^	0.70 ± 0.04 ^b^	80.36 ± 5.07 ^b^

Different letters indicate statistically significant differences between the coffee samples (*p* ≤ 0.05).

**Table 4:** Means and standard deviations of L*, C*, and h values of demucilated coffee in natura and coffee dried via different drying processes. Data expressed as means ± standard deviation (*n* = 10). Means followed by the same letters in the column do not differ at a 5% significance level, according to Tukey’s test.

	L*	C*	h
Method	Processed dry coffees		
Suspended terrace	38.04 ± 4.33 ^a^	11.45 ± 2.58 ^a^	88.86 ± 2.80 ^a^
Convective oven	42.03 ± 4.03 ^a^	13.39 ± 1.18 ^a^	88.34 ± 1.84 ^a^
Cast-tape dryer	43.72 ± 3.42 ^a^	13.94 ± 2.04 ^a^	88.20 ± 1.28 ^a^
Fixed-bed dryer	39.87 ± 5.52 ^a^	13.17 ± 1.54 ^a^	87.32 ± 2.44 ^a^
Rotary drum dryer	43.28 ± 4.92 ^a^	13.85 ± 1.60 ^a^	88.66 ± 1.05 ^a^
Combined drying	38.50 ± 2.77 ^a^	11.91 ± 1.53 ^a^	88.48 ± 1.82 ^a^

**Table 5:** Mean scores assigned by Q-graders of coffee dried via different drying processes. Data expressed as means ± standard deviation (*n* = 5). Means followed by the same letters in the column do not differ at a 5% significance level, according to Tukey’s test. ST: Suspended patio; CO: Convective dryer; CTD: Cast-tape dryer; FBD: Fixed-bed dryer; RDD: Rotary drum dryer; and CD: Combined industrial dryers.

Attribute	ST	CO	CTD	FBD	RDD	CD
Aroma	7.90 ± 0.42 ^a^	8.00 ± 0.40 ^a^	7.70 ± 0.21 ^a^	7.70 ± 0.27 ^a^	7.80 ± 0.27 ^a^	7.70 ± 0.33 ^a^
Flavor	7.90 ± 0.42 ^a^	8.20 ± 0.41 ^a^	7.85 ± 0.14 ^a^	7.80 ± 0.21 ^a^	7.80 ± 0.60 ^a^	7.70 ± 0.33 ^a^
Aftertaste	7.90 ± 0.42 ^a^	8.13 ± 0.35 ^a^	7.55 ± 0.11 ^a^	7.75 ± 0.18 ^a^	7.85 ± 0.52 ^a^	7.45 ± 0.41 ^a^
Acidity	8.10 ± 0.60 ^a^	8.25 ± 0.40 ^a^	7.95 ± 0.37 ^a^	7.70 ± 0.27 ^a^	7.85 ± 0.52 ^a^	7.80 ± 0.21 ^a^
Body	7.85 ± 0.42 ^a^	8.00 ± 0.31 ^a^	7.65 ± 0.29 ^a^	7.90 ± 0.22 ^a^	8.00 ± 0.47 ^a^	7.55 ± 0.21 ^a^
Balance	7.80 ± 0.45 ^a^	8.05 ± 0.45 ^a^	7.80 ± 0.11 ^a^	7.85 ± 0.52 ^a^	7.90 ± 0.34 ^a^	7.65 ± 0.34 ^a^
Overall	7.85 ± 0.55 ^a^	8.10 ± 0.29 ^a^	7.60 ± 0.22 ^a^	7.75 ± 0.25 ^a^	7.85 ± 0.34 ^a^	7.60 ± 0.29 ^a^
Total	85.30 ± 2.98 ^a^	86.75 ± 2.37 ^a^	84.10 ± 0.65 ^a^	84.45 ± 1.36 ^a^	85.05 ± 2.85 ^a^	83.45 ± 1.65 ^a^

## Data Availability

The original contributions presented in the study are included in the article, further inquiries can be directed to the corresponding author.
